# Diversity of trypanorhynch metacestodes in teleost fishes from coral reefs off eastern Australia and New Caledonia

**DOI:** 10.1051/parasite/2014060

**Published:** 2014-11-18

**Authors:** Ian Beveridge, Rodney A. Bray, Thomas H. Cribb, Jean-Lou Justine

**Affiliations:** 1 Veterinary Clinical Centre, University of Melbourne, Werribee Victoria 3030 Australia; 2 Department of Life Sciences, Natural History Museum Cromwell Road London SW7 5BD United Kingdom; 3 School of Biological Sciences, University of Queensland Brisbane Queensland 4072 Australia; 4 ISYEB, Institut de Systématique, Évolution, Biodiversité (UMR7205 CNRS, EPHE, MNHN, UPMC), Muséum National d’Histoire Naturelle CP 51 55 rue Buffon 75231 Paris Cedex 05 France

**Keywords:** Trypanorhyncha, Metacestodes, Great Barrier Reef, New Caledonia, Teleosts

## Abstract

Trypanorhynch metacestodes were examined from teleosts from coral reefs in eastern Australia and from New Caledonia. From over 12,000 fishes examined, 33 named species of trypanorhynchs were recovered as well as three species of tentacularioids which are described but not named. Host-parasite and parasite-host lists are provided, including more than 100 new host records. Lacistorhynchoid and tentacularioid taxa predominated with fewer otobothrioid and gymnorhynchoids. Five species, *Callitetrarhynchus gracilis*, *Floriceps minacanthus*, *Pseudotobothrium dipsacum*, *Pseudolacistorhynchus heroniensis* and *Ps. shipleyi*, were particularly common and exhibited low host specificity. Limited data suggested a higher diversity of larval trypanorhynchs in larger piscivorous fish families. Several fish families surveyed extensively (Blenniidae, Chaetodontidae, Gobiidae, Kyphosidae and Scaridae) yielded no trypanorhynch larvae. The overall similarity between the fauna of the Great Barrier Reef and New Caledonia was 45%. Where available, information on the adult stages in elasmobranchs has been included.

## Abstract (fr)

Les métacestodes de Trypanorhynques de téléostéens des récifs coralliens de l’est de l’Australie et de Nouvelle-Calédonie ont été examinés. À partir de plus de 12000 poissons examinés, 33 espèces nommées de Trypanorhynques ont été collectées ainsi que trois espèces de Tentacularioidea qui sont décrites mais non nommées. Des listes hôtes-parasites et parasites-hôtes sont fournies, et incluent plus de 100 nouvelles mentions d’hôtes. Les taxa appartenant aux Lacistorhynchoidea et Tentacularioidea prédominaient et les Otobothrioidea et Gymnorhynchoidea étaient moins nombreux. Cinq espèces, *Callitetrarhynchus gracilis*, *Floriceps minacanthus*, *Pseudotobothrium dipsacum*, *Pseudolacistorhynchus heroniensis* et *Ps. shipleyi* étaient particulièrement fréquentes et montraient une faible spécificité d’hôte. Des données limitées suggèrent une plus grande diversité de Trypanorhynques larvaires dans les familles de poissons piscivores de grande taille. Plusieurs familles de poissons étudiées intensivement (Blenniidae, Chaetodontidae, Gobiidae, Kyphosidae et Scaridae) n’avaient pas de larves de Trypanorhynques. La similitude globale entre les faunes de la Grande Barrière de Corail et de la Nouvelle-Calédonie était de 45 %. Des informations sur les stades adultes chez des élasmobranches ont été incluses quand disponibles.

## Introduction

The identification of significant threats to the coral reefs of the world [9, 17] has been partly responsible for focussing attention on the full diversity of reefs rather than simply on the diversity of fish and corals, the most obvious examples of reef diversity. The contributions of other groups of invertebrates to diversity on reefs have been largely overlooked in the past [7, 32]. Part of this “hidden” invertebrate diversity includes the endoparasites of vertebrates.

In recent years, teleost fish occurring on coral reefs have been recognised as harbouring a particularly diverse array of parasites [20]. Studies to date have focussed either on specific parasite groups such as the Monogenea (e.g. [33]) or Digenea (e.g. [13]), or more recently have examined the diversity of all helminth parasites found in or on specific families of fish such as the Lethrinidae or Serranidae [21–23].

Teleosts found on coral reefs are commonly infected with the larval stages (plerocerci, merocerci or plerocercoids – for terminology see Chervy, 2002 [12]) of cestodes of the order Trypanorhyncha, the adults of which are found in the stomach or spiral valves of elasmobranchs. Larval stages occur most commonly in the body cavity but may also be found in the musculature or other sites such as the gill arches [27]. They constitute a significant component of parasite diversity but have frequently been overlooked because of taxonomic difficulties in identification [27]. However, unlike other orders of cestodes found in marine fish, the larval stages have scolex features, including the distinctive tentacular armature, which are identical to those found in the adult and which allow specific morphological identification. Although taxonomic studies of this group of parasites are frequent, ecological studies are few, and while systematic collecting has been undertaken in several parts of the world (Gulf of Mexico, Gulf of California, Java, Borneo, Australia and Hawaii), there are few published descriptions of the faunas encountered in these areas (see Jensen, 2009 [19] for Gulf of Mexico and Palm and Bray, 2014 [29] for Hawaii). Some species of trypanorhynchs (e.g. *Grillotia* (*Christianella*) *minuta* van Beneden, 1858; *Gilquinia squali* Fabricius, 1794) have also been used as biological tags in teleosts [25] because the larval stages are readily identifiable and because they are long-lived in the intermediate host. However, such ecological studies of these species are limited.

In this study, we examined the larval trypanorhynch cestode parasites of teleosts, and where applicable the corresponding adults in elasmobranchs, from the Great Barrier Reef (GBR) and compared them with those from similar reef environments in New Caledonia (NC). New Caledonia is separated from the GBR by about 1200 km of deep oceanic waters.

## Materials and methods

### Great Barrier Reef (GBR)

**Figure 1.**
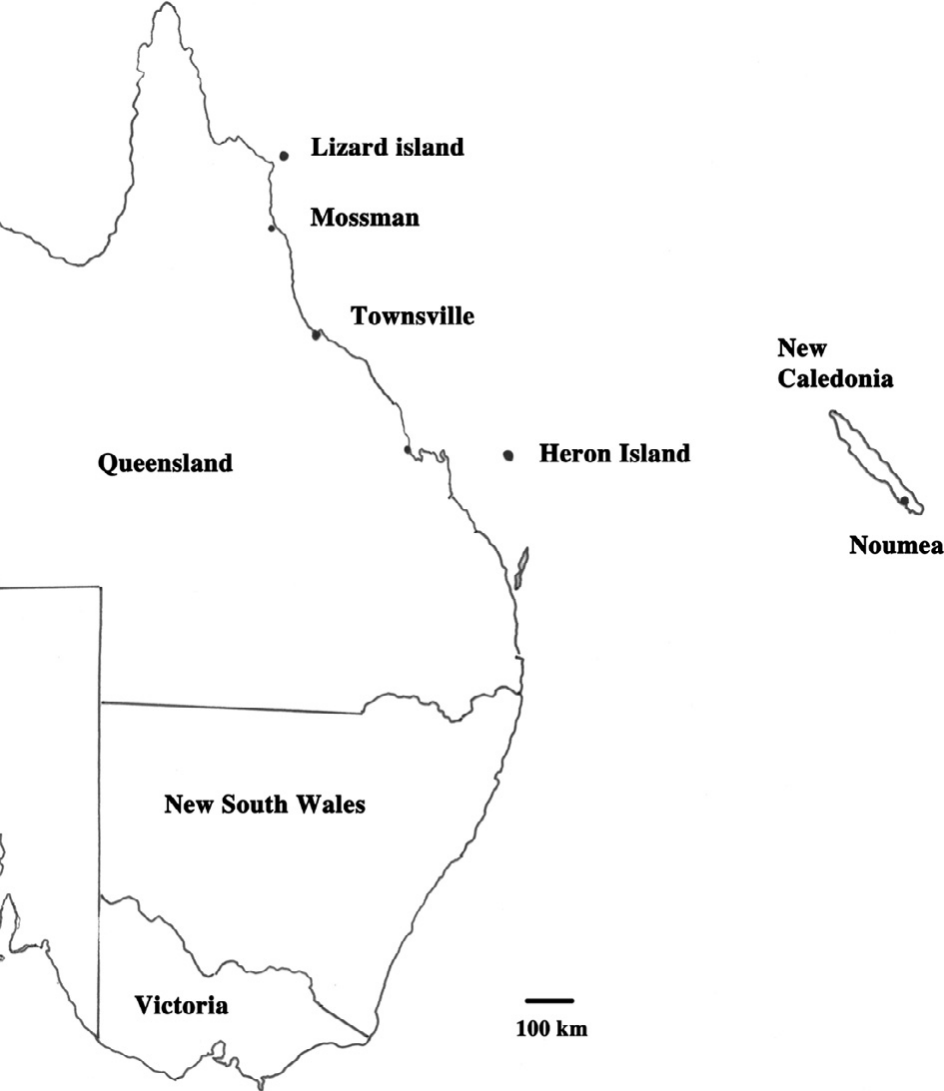
Collection localities off the east coast of Australia and New Caledonia.

Teleosts and elasmobranchs were collected opportunistically between 1986 and 2010. The two main collecting sites were Heron Island in the southern Great Barrier Reef and Lizard Island in the Northern Barrier Reef. Small numbers of parasites were collected on reefs between these two sites (Mossman, Townsville) and in these instances, the nearest geographical feature on the coast was recorded rather than the specific reef near which the collection was made (Fig. 1).

Metacestodes were collected mainly from body cavities of teleosts, although in some instances they were sought in regions of the body such as the gill arches and musculature. Metacestodes were removed from surrounding cysts (in the case of plerocerci) and the eversion of tentacles was achieved either by shaking vigorously or by applying pressure under a coverslip. Cestodes were fixed in 70% ethanol or 10% formalin and were stained with Celestine blue or carmine (Palm, 2004) [27], dehydrated in ethanol, cleared in methyl salicylate and mounted in Canada balsam. All specimens were identified by IB and have been deposited in either the British Museum (Natural History) (BMNH), the Queensland Museum, Brisbane (QM) or the South Australian Museum, Adelaide (SAM). Some of the records used in this compilation have been published previously in Beveridge & Campbell, 1996, 2001 [1, 3], Beveridge et al., 2000, 2007 [4, 5], Campbell & Beveridge, 1996 [8], Palm, 2004 [27], Palm & Beveridge, 2002 [28] and Sakanari, 1989 [34].

Records of adults from elasmobranchs are included only for species in which larval stages have been identified in teleosts; these are based on both published data and specimens held in museum collections. Additional species of trypanorhynch cestodes from elasmobranchs have been found and their larval stages may be found in the future, but for the present study, these records have not been added.

### New Caledonia (NC)

Fish were collected opportunistically between 2003 and 2009 generally by line fishing, occasionally by spear fishing and on occasions supplemented by fish obtained from a market. Collections were mainly off Nouméa (Fig. 1). All fish were measured, weighed and photographed. Methods for collection from several host families have been explained elsewhere [21–23]. Trypanorhynch plerocerci were opened and compressed between two slides or immersed in hot saline to evert tentacles. Plerocercoids found in the body cavity were also fixed under pressure to evert tentacles. Metacestodes were fixed in 70% ethanol or 10% formalin and were stained with Celestine blue or carmine [27], dehydrated in ethanol, cleared and mounted in Canada balsam. All specimens were identified by IB and have been deposited in the Muséum national d’Histoire naturelle, Paris (MNHN). Particular difficulties were encountered in the identification of tentaculariid cestodes from New Caledonia. Consequently, brief descriptions, some measurements and illustrations of each unidentified species encountered are included. Drawings were made with a drawing tube attached to an Olympus BH 2 microscope. Representative, rather than comprehensive, measurements were made with an ocular micrometer and are presented in micrometers.

In the parasite-host list (Table 1), authorities of cestodes are included and host species are listed in alphabetical order without authorities. In instances where both generic and specific names of cestodes have changed, synonyms have been included. In the host-parasite list (Table 2), fish hosts are arranged in orders, families and genera, but within each group, the order is alphabetical. Authorities of fish are indicated and the parasites are arranged in alphabetical order without authorities.

**Table 1.** Parasite-host list. Species of trypanorhynch cestodes collected from teleosts and elasmobranchs on the Great Barrier Reef, Australia and from New Caledonia. Authorities of cestodes are included and host species are listed in alphabetical order without authorities.

	Great Barrier Reef	New Caledonia	
GYMNORHYNCHOIDEA			
***Molicola horridus*** (Goodsir, 1841)			
Larval			
*Diodon hystrix*	H^A^ QM^C^ G206954, SAM^E^ 44079	*Diodon hystrix*	MNHN^D^ JNC2977D1, 3199C
*Diodon liturosus* *	L^B^ QM G232552		
***Pterobothrium lintoni*** MacCallum, 1916			
Larval			
*Choerodon venustus*	H SAM 40480		
***Pterobothrium acanthotruncatum***			
Escalante & Carvajal, 1984			
Larval			
Plectropomus maculatus*	H QM G217640		
*Scomberomorus commerson*	H, L QM G217628		
Adult			
Pristis zijsron*	Tv^G^ SAM 35749		
***Pterobothrium australiense*** Campbell & Beveridge, 1996			
Larval			
Halichoeres trimaculatus*	H QM G217629		
Adult			
*Pristis zijsron*	Tv SAM 23898		
***Pterobothrium pearsoni*** (Southwell, 1929)			
Larval			
*Sphyraena jello* *	L QM G233646		
LACISTORHYNCHOIDEA			
***Bombycirhynchus sphaerenaicum*** (Pintner, 1930)			
Larval			
Sphyraena jello*	L QM G233583		
***Callitetrarhynchus gracilis*** (Rudolphi, 1819)			
Larval			
Abudefduf whitleyi*	H QM G212162	Atule mate*	MNHN JNC2814T, 2963, 2964, 2965, 3371
Apogon poecilopterus*	H QM G217587	Carangoides fulvoguttatus*	MNHN JNC463C
Caesio cuning*	H QM G217593	Caranx papuensis*	MNHN JNC1189E
*Cephalopholis miniata*	H QM G232625	*Cephalopholis boenak*	[20]^J^
Cephalopholis cyanostigma*	H, L QM G217575	Cephalopholis spiloparaea*	MNHN JNC2624
Choerodon cyanodus*	H BM 1980.7.10.148–9	*Chirocentrus dorab*	MNHN JNC3220
Cromileptes altivelis*	H QM G217592	*Epinephelus chlorostigma*	MNHN JNC2446C
Johnius borneensis*	H QM G217602	Epinephelus fasciatus*	MNHN JNC1256A, 2625, 3039
Lotella rhacina*	H QM G217574	Epinephelus retouti*	MNHN JNC3083
Lutjanus carponotatus*	L QM G233588	*Epinephelus rivulatus*	MNHN JNC2606C
Naso vlamingii*	H QM G217598	Lethrinus miniatus*	MNHN JNC2113A
Ostorhinchus fasciatus*	H QM G217486	*Lutjanus vitta*	[22]^J^
Plectropomus maculatus*	H QM G217641	*Megalaspis cordyla*	MNHN JNC1186, 1188
Polynemus heptadactyla*	H QM G217591	*Nemipterus furcosus*	MNHN JNC2596
*Pomatomus saltatrix*	H QM G217583	*Scomberomorus commerson*	MNHN JNC435
*Scomberomorus commerson*	H, L QM G212163	Triodon macropterus*	MNHN JNC2984
Scomberomorus queenslandicus*	H QM G217588	*Variola louti*	[20]^J^
Sphyraena obtusata*	H QM G217590		
Adult			
*Carcharhinus melanopterus*	H QM G217581	*Carcharhinus leucas*	MNHN JNC2856
*Carcharhinus amblyrhynchoides*	Si^H^ SAM 24941		
***Callitetrarhynchus speciosus*** (Linton, 1897)			
Larval		Cymbacephalus beauforti*	MNHN JNC1833
***Dasyrhynchus basipunctatus*** (Carvajal, Campbell & Cornford, 1976)			
Fistularia commersonii*	H QM G232633	Abalistes filamentosus*	MNHN JNC2193
		*Abalistes stellatus*	MNHN JNC2163, 2926, 2914
		*Diodon hystrix*	MNHN JNC2977
		Lagocephalus sceleratus*	MNHN JNC2942
		Pseudobalistes fuscus*	MNHN JNC1680E, 2164, 2940
		Triodon macropterus*	MNHN JNC2989
Adult			
*Carcharhinus brachyurus*	L QM G232540	*Carcharhinus amblyrhynchos*	MNHN JNC435, 1111
Carcharhinus melanopterus*	H QM G232634	Carcharhinus plumbeus*	MNHN JNC442
***Diesingium* cf *lomentaceum*** (Diesing, 1850)			
Larval			
		Carangoides fulvoguttatus*	MNHN JNC3169
		Epinephelus chlorostigma*	MNHN JNC3142
***Floriceps minacanthus*** Campbell & Beveridge, 1987			
Larval			
Cephalopholis boenak*	H QM G212151–3	*Cephalopholis miniata*	MNHN JNC2627
Cephalopholis cyanostigma*	L QM G233613	Cephalopholis sonnerati*	MNHN JNC2934, 2935, 2936, 3029
*Cephalopholis miniata*	H QM G217615	*Cephalopholis urodeta*	[20]^J^
*Epinephelus quoyanus*	H SAM 44083	*Epinephelus coioides*	MNHN JNC3257
*Euthynnus affinis*	H QM G217612, 7	*Epinephelus cyanopodus*	MNHN JNC1998
Euthynnus alletteratus*	H SAM 44082	Epinephelus maculatus*	MNHN JNC2937, 3061, 3062, 3066
Grammatorcynus bicarinatus*	H, L QM G217613	Lethrinus miniatus*	MNHN JNC2706A
Lethrinus miniatus*	H QM G233554	*Nemipterus furcosus*	MNHN JNC3019
Plectropomus areolatus*	L QM G233626	*Plectropomus leopardus*	MNHN JNC2585A
*Plectropomus leopardus*	H, L QM G217611, SAM 32139	*Plectropomus laevis*	MNHN JNC1887
Sphyraena flavicauda*	H QM G217616	Sphyraena putnamae*	MNHN JNC3035
Sphyraena jello*	L QM G233610	Tylosurus crocodilus*	MNHN JNC1262C, 1263A
Tylosurus crocodilus*	H QM G217614	*Variola louti*	MNHN JNC1859B, 3037
Adult			
*Carcharhinus amboinensis*	StL^I^ SAM 22652	Carcharhinus leucas*	MNHN
		Triaenodon obesus*	MNHN
***Floriceps saccatus*** (Cuvier, 1817)			
Larval			
*Diodon hystrix*	H SAM 44081	Caranx papuensis*	MNHN JNC3209
Diodon liturosus*	L QM G232554	*Diodon hystrix*	MNHN JNC2343, 2977, 3199
***Grillotiella exile*** (Linton, 1909)			
Larval			
*Scomberomorus commerson*	L QM G233632		
Adult		*Galeocerdo cuvier*	MNHN JNC1414
***Microbothriorhynchus coelorhynchi*** Yamaguti, 1952			
Larval		Conger cinereus*	MNHN JNC2993
***Pseudogilquinia microbothria*** (MacCallum, 1917)			
(= *Ps. magna*; = *Dasyrhynchus magnus*)			
Larval			
Lethrinus atkinsoni*	L QM G233653	Lethrinus miniatus*	MNHN JNC2113 B1, 2158C
Lethrinus nebulosus*	L QM G233654		
***Pseudogilquinia pillersi*** (Southwell, 1929)			
Larval			
*Lethrinus atkinsoni*	H BM^F^ 2004.3.18.98–99	*Epinephelus coioides*	MNHN JNC1535, 3140, 3265B
*Lethrinus miniatus*	H BM 2004.3.18.97	*Plectropomus laevis*	MNHN JNC1865, 1887
*Lethrinus nebulosus*	L QM G233653	*Epinephelus malabaricus*	MNHN JNC1536
***Pseudolacistorhynchus heroniensis*** (Sakanari, 1989)			
Larval			
Cephalopholis miniata*	H QM G212146	Abalistes filamentosus*	MNHN JNC2724
*Epinephelus fasciatus*	H QM G217518, SAM 17418	Abalistes stellatus*	MNHN JNC2163, 2914, 2926
Epinephelus ongus*	H QM G214949	*Cephalopholis boenak*	MNHN JNC2889, 2890, 3205
*Epinephelus quoyanus*	H QM G212157, SAM 28629	Cephalopholis sonnerati*	MNHN JNC2934
Lethrinus miniatus*	H QM G212154	Gymnocranius grandoculis*	MNHN JNC1726
*Lethrinus nebulosus*	H [24]^J^	*Epinephelus chlorostigma*	MNHN JNC2446C, 3141
*Plectropomus leopardus*	H QM G212158, SAM 28681	Epinephelus coioides*	MNHN JNC3257
		*Epinephelus cyanopodus*	[20]^J^
		*Epinephelus fasciatus*	MNHN JNC1636A, 1758, 1791, 1792, 3039
		Epinephelus howlandi*	MNHN JNC2768
		*Epinephelus polyphekadion*	MNHN JNC1915C, 3036
		*Epinephelus rivulatus*	MNHN JNC1545C
		Lethrinus miniatus*	MNHN JNC2161C
		Lutjanus vitta*	[22]^J^
		*Plectropomus leopardus*	MNHN JNC3279
		Pseudobalistes fuscus*	MNHN JNC2164, 2940B
Adult		*Stegostoma fasciatum*	MNHN JNC1529
***Pseudolacistorhynchus shipleyi*** (Southwell, 1929)			
(= *Grillotia overstreeti* Sakanari, 1989)			
Larval			
Cephalopholis boenak*	H QM G232626	Cephalopholis sonnerati*	MNHN JNC3032
Cephalopholis cyanostigma*	H, L QM G214957	*Cephalopholis urodeta*	[20]^J^
*Choerodon cyanodus*	H SAM 17416, QM G212160	Epinephelus polyphekadion*	MNHN JNC3036
Choerodon fasciatus*	H QM G217519	Sufflamen fraenatus*	MNHN JNC1421C, 1797, 1798A, 1946, 2928, 3034
Epinephelus ongus*	H QM G212161	Epinephelus ongus*	MNHN JNC3275
Lotella rhacina*	H QM G214995		
Rhinecanthus aculeatus*	L QM G232542		
Sufflamen fraenatus*	H QM G217520		
OTOBOTHRIOIDEA			
***Otobothrium alexanderi*** Palm, 2004			
Larval			
*Tylosurus crocodilus*	L QM G232555	*Tylosurus crocodilus*	MNHN JNC1968
***Otobothrium parvum*** Beveridge & Justine, 2007			
Larval		Epinephelus maculatus*	MNHN JNC1405
		Lethrinus rubrioperculatus*	MNHN JNC1635A
Adult		*Carcharhinus amblyrhynchos*	MNHN JNC1111
		*Triaenodon obesus*	MNHN JNC2109
***Otobothrium penetrans*** Linton, 1907			
Larval†		*Tylosurus crocodilus*	MNHN JNC1968
***Proemotobothrium southwelli*** Beveridge & Campbell, 2001			
Larval			
*Johnius borneensis*	H QM G217939		
***Pseudotobothrium dipsacum*** (Linton, 1897)			
Larval			
*Abalistes stellatus*	H QM G217928–32	Abalistes filamentosus*	MNHN JNC2724
*Cephalopholis cyanostigma*	H QM G214959	*Abalistes stellatus*	MNHN JNC2914
*Cheilinus trilobatus*	L QM G233555	Cephalopholis miniata*	MNHN JNC2627
*Epinephelus coioides*	Tv SAM 31342	Cephalopholis sonnerati*	MNHN JNC1616, 2934–6
*Lethrinus obsoletus*	H QM G233888	*Cephalopholis urodeta*	MNHN JNC2750
*Lutjanus gibbus*	L QM GL 10508	Cymbacephalus beauforti*	MNHN JNC1833A
*Naso vlamingii*	H QM G214960	*Epinephelus coioides*	MNHN JNC1535, 3257
*Plectropomus leopardus*	H, L QM G217936	Epinephelus fasciatus*	MNHN JNC1791, 3039
*Plectropomus maculatus*	H QM G206964	*Epinephelus malabaricus*	[20]^J^
*Pseudocaranx dentex*	H QM G214961	Epinephelus retouti*	MNHN JNC2179
*Rhinecanthus aculeatus*	L QM G232590	Plectropomus laevis*	MNHN JNC1865, 1887
*Rhinecanthus rectangulus*	H QM G217934	*Plectropomus leopardus*	MNHN JNC2126
		Pseudobalistes fuscus*	MNHN JNC2927, 2940
		*Variola louti*	MNHN JNC1629, 1662, 1756–7, 1859, 2116–7, 2301, 3037, 3069
***Symbothriorhynchus tigaminacanthus*** Palm, 2004			
Larval		Nemipterus furcosus*	MNHN JNC2586, 2610
		Saurida undosquamis*	MNHN JNC2079
Adult		*Sphyrna lewini*	MNHN JNC1628
TENTACULARIOIDEA			
***Hepatoxylon trichiuri***			
Larval			
Diodon hystrix*	H QM G227128	Diodon hystrix*	MNHN JNC2977, 3199D
		Tetrapterus angustirostris*	MNHN JNC1399
		Thunnus obesus*	MNHN JNC1398
			
Adult†		Prionace glauca*	MNHN JNC1217
***Heteronybelinia estigmena*** (Dollfus, 1960)			
Larval			
*Sarda australis*	H QM G218042–6	Atule mate*	MNHN JNC2963–5
		Herklotsichthys quadrimaculatus*	MNHN JNC2669B, 2673, 2943, 2949
		*Selar crumenophthalmus*	MNHN JNC3043–4, 3126
		Sphyraena putnamae*	MNHN JNC3035
		Trichiurus lepturus*	MNHN JNC3045–6, 3048
Adult			
*Carcharhinus* sp.	Qld SAM 18322	*Carcharhinus brevipinna*	MNHN JNC3138
***Heteronybelinia* sp. C**			
Larval		*Sufflamen fraenatus*	MNHN JNC3034
***Myxonybelinia southwelli*** (Palm & Walter, 1999)			
Larval			
*Choerodon venustus*	H QM G218062		
Adult		*Stegostoma fasciatum*	MNHN JNC1529
***Nybelinia aequidentata*** Shipley & Hornell, 1906			
Larval			
		Dendrochirus zebra*	QM G218031
***Nybelinia basimegacantha*** Carvajal, Campbell & Cornford, 1976			
Larval			
Parupeneus bifasciatus*	L QM G232545	Neoniphon sammara*	MNHN JNC2552
		*Parupeneus multifasciatus*	MNHN JNC2111
***Nybelinia goreensis*** Dollfus, 1960			
Larval		Lethrinus genivittatus*	MNHN JNC2033
		Lethrinus rubrioperculatus*	MNHN JNC1148
		*Nemipterus furcosus*	MNHN JNC2612
		Parupeneus barberinus*	MNHN JNC1838B
		Parupeneus multifasciatus*	MNHN JNC2112
***Nybelinia indica*** Chandra, 1986			
(= *Nybelinia scoliodoni* Vijayalakshmi, Vijayalakshmi & Gangadharam, 1996)			
Larval			
*Diodon hystrix*	H QM G218034–41	*Caranx sexfasciatus*	MNHN JNC3194
		*Diodon hystrix*	MNHN JNC2977F
		Lagocephalus sceleratus*	MNHN JNC2982
		Leiognathus fasciatus*	MNHN JNC2921
		Nemipterus furcosus*	MNHN JNC2288, 2611, 3016
Adult			
*Taeniura lymma*	H SAM 17646	Triaenodon obesus*	MNHN JNC2109B1
***Nybelinia queenslandensis*** Jones & Beveridge, 1998			
Larval			
Ostorhinchus cookii*	H QM G232539	Nemipterus furcosus*	MNHN JNC3011–2
Ostorhinchus properuptus*	L QM G2336644		
Adult			
*Carcharhinus melanopterus*	H, L QM G217521–31		
***Nybelinia strongyla*** Dollfus, 1960			
Larval			
*Johnius borneensis*	H QM G218109		
***Nybelinia* sp. A**			
Larval		*Herklotsichthys quadrimaculatus*	MNHN JNC2669C
***Nybelinia* sp. B**			
Larval		*Parupeneus multifasciatus*	MNHN JNC2172C

† Reported in the literature from Australia but outside the region of the Great Barrier Reef.

* New host records.

A Heron Island, Great Barrier Reef.

B Lizard Island, Great Barrier Reef.

C Queensland Museum, Brisbane.

D Muséum national d’Histoire naturelle, Paris.

E South Australian Museum, Adelaide.

F British Museum, Natural History, London.

G Townsville, Queensland.

H Snapper Island, Mossman.

I St Lawrence, Queensland.

J Published report not supported by museum specimen.

**Table 2.** Species of trypanorhynch cestodes collected from teleosts on the Great Barrier Reef, Australia and from New Caledonia. Authorities of fish are included and cestodes are listed in alphabetical order without authorities. GBR: Great Barrier Reef; NC: New Caledonia.

Order	Family	Host species	Parasites	Location
Anguilliformes	Congridae	*Conger cinereus* Rüppell, 1830	*Microbothriorhynchus coelorhynchi*	NC
Aulopiformes	Synodontidae	*Saurida undosquamis* (Richardson, 1848)	*Symbothriorhynchus tigaminacanthus*	NC
Beloniformes	Belonidae	*Tylosurus crocodilus* (Péron & Lesueur, 1821)	*Floriceps minacanthus*	GBR, NC
			*Otobothrium alexanderi*	GBR, NC
			*Otobothrium penetrans*	NC
Beryciformes	Holocentridae	*Neoniphon sammara* (Forsskål, 1775)	*Nybelinia basimegacantha*	NC
Clupeiformes	Chirocentridae	*Chirocentrus dorab* (Forsskål, 1775)	*Callitetrarhynchus gracilis*	NC
	Clupeidae	*Herklotsichthys quadrimaculatus* (Rüppell, 1837)	*Heteronybelinia estigmena*	NC
			*Nybelinia* sp. A	NC
Gadiformes	Moridae	*Lotella rhacina* (Forster, 1801)	*Callitetrarhynchus gracilis*	GBR
			*Pseudolacistorhynchus shipleyi*	GBR
Perciformes	Acanthuridae	*Naso vlamingii* (Valenciennes, 1835)	*Callitetrarhynchus gracilis*	GBR
			*Pseudotobothrium dipsacum*	GBR
	Apogonidae	*Apogon poecilopterus* Cuvier, 1828	*Callitetrarhynchus gracilis*	GBR
		*Ostorhinchus cookii* (Macleay, 1881)	*Nybelinia queenslandensis*	GR
		*Ostorhinchus fasciatus* (White, 1790)	*Callitetrarhynchus gracilis*	GBR
		*Ostorhinchus properuptus* (Whitley, 1964)	*Nybelinia queenslandensis*	GBR
	Carangidae	*Atule mate* (Cuvier, 1833)	*Callitetrarhynchus gracilis*	NC
			*Heteronybelinia estigmena*	NC
		*Carangoides fulvoguttatus* (Forsskål, 1775)	*Callitetrarhynchus gracilis*	NC
			*Diesingium* cf *lomentaceum*	NC
		*Carangoides sexfasciatus* Quoy & Gaimard, 1825	*Nybelinia indica*	NC
		*Caranx papuensis* Alleyne & Macleay, 1877	*Callitetrarhynchus gracilis*	NC
			*Floriceps saccatus*	NC
		*Megalaspis cordyla* (Linnaeus, 1758)	*Callitetrarhynchus gracilis*	NC
		*Pseudocaranx dentex* (Bloch & Schneider, 1801)	*Pseudotobothrium dipsacum*	GBR
		*Selar crumenophthalmus* (Bloch, 1793)	*Heteronybelinia estigmena*	NC
		*Tetrapterus angustirostris* Tanka, 1915	*Hepatoxylon trichiuri*	NC
	Labridae	*Cheilinus trilobatus* (Lacépède, 1801)	*Pseudotobothrium dipsacum*	GBR
		*Halichoeres trimaculatus* (Quoy & Gaimard, 1834)	*Pterobothrium australiense*	GBR
		*Choerodon cyanodus* (Richardson, 1843)	*Callitetrarhynchus gracilis*	GBR
			*Pseudolacistorhynchus shipleyi*	GBR
		*Choerodon fasciatus* (Günther, 1867)	*Pseudolacistorhynchus shipleyi*	GBR
		*Choerodon venustus* (De Vis, 1884)	*Myxonybelinia southwelli*	GBR
			*Pterobothrium lintoni*	GBR
	Leiognathidae	*Leiognathus fasciatus* (Lacépède, 1803)	*Nybelinia indica*	NC
	Lethrinidae	*Lethrinus atkinsoni* Seale, 1910	*Pseudogilquinia microbothria*	GBR
			*Pseudogilquinia pillersi*	GBR
		*Lethrinus genivittatus* Valenciennes, 1830	*Nybelinia goreensis*	NC
		*Lethrinus miniatus* (Forster, 1801)	*Callitetrarhynchus gracilis*	NC
			*Floriceps minacanthus*	GBR, NC
			*Pseudolacistorhynchus heroniensis*	GBR, NC
			*Pseudogilquinia microbothria*	NC
			*Pseudogilquinia pillersi*	GBR
		*Lethrinus nebulosus* (Forsskål, 1775)	*Pseudogilquinia microbothria*	GBR
			*Pseudogilquinia pillersi*	GBR
			*Pseudolacistorhynchus heroniensis*	GBR
		*Lethrinus obsoletus* (Forsskål, 1775)	*Pseudotobothrium dipsacum*	GBR
		*Lethrinus rubrioperculatus* Sato, 1978	*Nybelinia goreensis*	NC
			*Otobothrium parvum*	NC
		*Gymnocranius grandoculis* (Valenciennes, 1830)	*Pseudolacistorhynchus heroniensis*	NC
	Lutjanidae	*Caesio cuning* (Bloch, 1791)	*Callitetrarhynchus gracilis*	GBR
		*Lutjanus carponotatus* (Richardson, 1842)	*Callitetrarhynchus gracilis*	GBR
		*Lutjanus gibbus* (Forsskål, 1775)	*Pseudotobothrium dipsacum*	GBR
		*Lutjanus vitta* (Quoy & Gaimard, 1824)	*Callitetrarhynchus gracilis*	NC
			*Pseudolacistorhynchus heroniensis*	NC
	Mullidae	*Parupeneus barberinus* (Lacépède, 1801)	*Nybelinia goreensis*	NC
		*Parupeneus bifasciatus* (Lacépède, 1801)	*Nybelinia basimegacantha*	GBR
		*Parupeneus multifasciatus* (Quoy & Gaimard, 1825)	*Nybelinia basimegacantha*	NC
			*Nybelinia goreensis*	NC
			*Nybelinia* sp. B	NC
	Nemipteridae	*Nemipterus furcosus* (Valenciennes, 1830)	*Callitetrarhynchus gracilis*	NC
			*Floriceps minacanthus*	NC
			*Nybelinia indica*	NC
			*Nybelinia goreensis*	NC
			*Nybelinia queenslandensis*	NC
			*Symbothriorhynchus tigaminacanthus*	NC
	Polynemidae	*Polynemus heptadactyla* (Cuvier, 1829)	*Callitetrarhynchus gracilis*	GBR
	Pomacentridae	*Abudefduf whitleyi* Allen & Robertson, 1974	*Callitetrarhynchus gracilis*	GBR
	Pomatomidae	*Pomatomus saltatrix* (Linnaeus, 1766)	*Callitetrarhynchus gracilis*	GBR
	Sciaenidae	*Johnius borneensis* (Bleeker, 1851)	*Callitetrarhynchus gracilis*	GBR
			*Nybelinia strongyla*	GBR
			*Proemotobothrium southwelli*	GBR
	Scombridae	*Euthynnus affinis* (Cantor, 1849)	*Floriceps minacanthus*	GBR
		*Euthynnus alletteratus* (Rafinesque, 1810)	*Floriceps minacanthus*	GBR
		*Grammatorcynus bicarinatus* (Quoy & Gaimard, 1825)	*Floriceps minacanthus*	GBR
		*Sarda australis* (Macleay, 1881)	*Heteronybelinia estigmena*	GBR
		*Scomberomorus commerson* (Lacépède, 1800)	*Callitetrarhynchus gracilis*	GBR, NC
			*Grillotiella exile*	GBR
			*Pterobothrium acanthotruncatum*	GBR
		*Scomberomorus queenslandicus* Munro, 1943	*Callitetrarhynchus gracilis*	GBR
		*Thunnus obesus* (Lowe, 1839)	*Hepatoxylon trichiuri*	NC
	Serranidae	*Cephalopholis boenak* (Bloch, 1790)	*Callitetrarhynchus gracilis*	NC
			*Floriceps minacanthus*	GBR
			*Pseudolacistorhynchus heroniensis*	NC
			*Pseudolacistorhynchus shipleyi*	GBR
		*Cephalopholis cyanostigma* (Valenciennes, 1828)	*Pseudolacistorhynchus shipleyi*	GBR
			*Callitetrarhynchus gracilis*	GBR
			*Floriceps minacanthus*	GBR
			*Pseudotobothrium dipsacum*	GBR
		*Cephalopholis miniata* (Forsskål, 1775)	*Callitetrarhynchus gracilis*	GBR
			*Floriceps minacanthus*	GBR, NC
			*Pseudolacistorhynchus heroniensis*	GBR
			*Pseudotobothrium dipsacum*	NC
		*Cephalopholis sonnerati* (Valenciennes, 1828)	*Floriceps minacanthus*	NC
			*Pseudolacistorhynchus heroniensis*	NC
			*Pseudotobothrium dipsacum*	NC
		*Cephalopholis spiloparaea* (Valenciennes, 1828)	*Callitetrarhynchus gracilis*	NC
		*Cephalopholis urodeta* (Schneider, 1801)	*Floriceps minacanthus*	NC
			*Pseudolacistorhynchus shipleyi*	NC
			*Pseudotobothrium dipsacum*	NC
		*Cromileptes altivelis* (Valenciennes, 1828)	*Callitetrarhynchus gracilis*	GBR
		*Epinephelus coioides* (Hamilton, 1822)	*Dasyrhynchus pacificus*	NC
			*Floriceps minacanthus*	NC
			*Pseudogilquinia pillersi*	NC
			*Pseudolacistorhynchus heroniensis*	NC
			*Pseudotobothrium dipsacum*	GBR, NC
		*Epinephelus chlorostigma* (Valenciennes, 1828)	*Callitetrarhynchus gracilis*	NC
			*Dasyrhynchus pacificus*	NC
			*Diesingium* cf *lomentaceum*	NC
			*Pseudolacistorhynchus heroniensis*	NC
		*Epinephelus cyanopodus* (Richardson, 1846)	*Floriceps minacanthus*	NC
			*Pseudolacistorhynchus heroniensis*	NC
		*Epinephelus fasciatus* (Forsskål, 1775)	*Callitetrarhynchus gracilis*	NC
			*Pseudolacistorhynchus heroniensis*	GBR, NC
			*Pseudotobothrium dipsacum*	NC
		*Epinephelus howlandi* (Günther, 1873)	*Pseudolacistorhynchus heroniensis*	NC
		*Epinephelus maculatus* (Bloch, 1790)	*Floriceps minacanthus*	NC
			*Otobothrium parvum*	NC
		*Epinephelus malabaricus* (Bloch & Schneider, 1801)	*Pseudogilquinia pillersi*	NC
			*Pseudotobothrium dipsacum*	NC
		*Epinephelus ongus* Bloch, 1793	*Pseudolacistorhynchus heroniensis*	GBR
			*Pseudolacistorhynchus shipleyi*	GBR, NC
		*Epinephelus polyphekadion* (Bleeker, 1849)	*Pseudolacistorhynchus heroniensis*	NC
			*Pseudolacistorhynchus shipleyi*	NC
		*Epinephelus retouti* (Bleeker, 1868)	*Pseudotobothrium dipsacum*	NC
			*Callitetrarhynchus gracilis*	NC
		*Epinephelus quoyanus* (Valenciennes, 1830)	*Floriceps minacanthus*	GBR
			*Pseudolacistorhynchus heroniensis*	GBR
		*Epinephelus rivulatus* (Valenciennes, 1830)	*Callitetrarhynchus gracilis*	NC
			*Pseudolacistorhynchus heroniensis*	NC
		*Plectropomus areolatus* (Rüppell, 1830)	*Floriceps minacanthus*	GBR
		*Plectropomus laevis* (Lacépède, 1801)	*Floriceps minacanthus*	NC
			*Pseudogilquinia pillersi*	NC
			*Pseudotobothrium dipsacum*	NC
		*Plectropomus leopardus* (Lacépède, 1802)	*Floriceps minacanthus*	GBR, NC
			*Pseudolacistorhynchus heroniensis*	GBR, NC
			*Pseudotobothrium dipsacum*	GBR, NC
		*Plectropomus maculatus* (Bloch, 1790)	*Callitetrarhynchus gracilis*	GBR
			*Pseudotobothrium dipsacum*	GBR
			*Pterobothrium acanthotruncatum*	GBR
		*Variola louti* (Forsskål, 1775)	*Callitetrarhynchus gracilis*	NC
			*Floriceps minacanthus*	NC
			*Pseudotobothrium dipsacum*	NC
	Sphyraenidae	*Sphyraena flavicauda* (Rüppell, 1838)	*Floriceps minacanthus*	GBR
		*Sphyraena jello* Cuvier, 1829	*Bombycirhynchus sphaerenaicum*	GBR
			*Floriceps minacanthus*	GBR
			*Pterobothrium pearsoni*	GBR
		*Sphyraena obtusata* Cuvier, 1829	*Callitetrarhynchus gracilis*	GBR
		*Sphyraena putnamae* Jordan & Seale, 1905	*Floriceps minacanthus*	NC
			*Heteronybelinia estigmena*	NC
Syngnathiformes	Fistulariidae	*Fistularia commersonii* Rüppell, 1838	*Dasyrhynchus basipunctatus*	GBR
	Trichiuridae	*Trichiurus lepturus* Linnaeus, 1758	*Heteronybelinia estigmena*	NC
Tetraodontiformes	Balistidae	*Abalistes filamentosus* Matsuura & Yoshino, 2004	*Dasyrhynchus basipunctatus*	NC
			*Pseudolacistorhynchus heroniensis*	NC
			*Pseudotobothrium dipsacum*	NC
		*Abalistes stellatus* (Anonymous, 1798)	*Dasyrhynchus basipunctatus*	NC
			*Pseudolacistorhynchus heroniensis*	NC
			*Pseudotobothrium dipsacum*	GBR, NC
		*Pseudobalistes fuscus* (Bloch & Schneider, 1801)	*Dasyrhynchus basipunctatus*	NC
			*Pseudolacistorhynchus heroniensis*	NC
			*Pseudotobothrium dipsacum*	NC
		*Rhinecanthus aculeatus* (Linnaeus, 1758)	*Pseudolacistorhynchus shipleyi*	GBR
			*Pseudotobothrium dipsacum*	GBR
		*Rhinecanthus rectangulus* (Bloch & Schneider, 1801)	*Pseudotobothrium dipsacum*	GBR
		*Sufflamen fraenatus* (Latreille, 1804)	*Heteronybelinia* sp. C	NC
			*Pseudolacistorhynchus shipleyi*	GBR, NC
	Diodontidae	*Diodon hystrix* Linnaeus, 1758	*Floriceps saccatus*	GBR, NC
			*Hepatoxylon trichiuri*	GBR, NC
			*Molicola horridus*	GBR, NC
			*Nybelinia indica*	GBR, NC
		*Diodon liturosus* Shaw, 1804	*Floriceps saccatus*	GBR
			*Molicola horridus*	GBR
			*Dasyrhynchus basipunctatus*	NC
	Tetraodontidae	*Lagocephalus sceleratus* (Gmelin, 1789)	*Nybelinia indica*	NC
			*Dasyrhynchus basipunctatus*	NC
		*Triodon macropterus* Lesson, 1831	*Callitetrarhynchus gracilis*	NC
			*Dasyrhynchus basipunctatus*	NC
Scorpaeniformes	Platycephalidae	*Cymbacephalus beauforti* (Knapp, 1973)	*Callitetrarhynchus speciosus*	NC
			*Pseudotobothrium dipsacum*	NC
	Scorpaenidae	*Dendrochirus zebra* (Cuvier, 1829)	*Nybelinia aequidentata*	NC

Authorities of hosts or parasites which are indicated in the lists are not repeated in the text. The systematic arrangement of trypanorhynch taxa follows Palm (2004) [27]. All host names were verified in FishBase [15].

## Results

### Species found and other data

**Figures 2–7.**
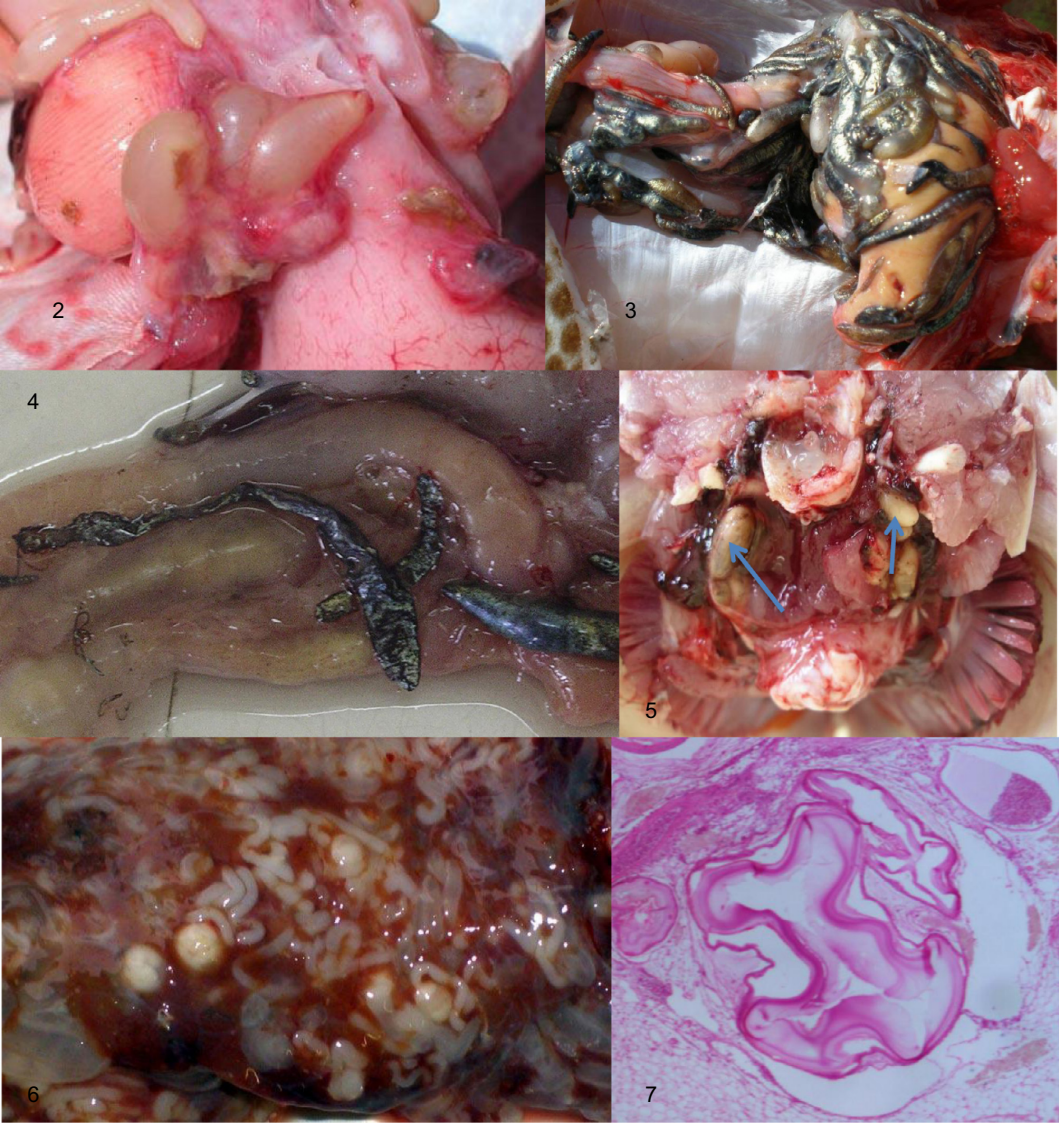
Metacestodes of trypanorhynch cestodes from teleost fishes. **2.** Viable plerocerci of *Callitetrarhynchus gracilis* in the body cavity of *Scomberomorus commerson*. **3**. Melanised trypanorhynch plerocerci in the body cavity of *Epinephelus* sp. **4**. Melanised and contracted cysts of trypanorhynch metacestodes in the body cavity of *Cephalopholis miniata*; no viable plerocerci were recovered from these cysts. **5**. Plerocerci of *Pseudogilquinia* spp. (arrows) around the oesophagus of *Lethrinus nebulosus*. **6**. Merocerci of *Molicola horridus* in the liver of *Diodon hystrix*. **7**. Plerocerci of *Grillotiella exile* in the gill arches of *Scomberomorus commerson* (histological section).

Larval trypanorhynchs were recovered primarily from the body cavities of the teleosts examined (Figs. 2–7). Plerocerci were usually encountered attached to the mesentery enclosed within white envelopes (Fig. 2), although in some hosts melanisation of the cyst wall had occurred rendering the cysts brown (Fig. 3). Some brown or even black envelopes contained only remnants of plerocerci (Fig. 4). Plerocercoids of tentaculariids were found either in the body cavity or in the gastrointestinal lumen; the latter were not contained within a “cyst”. Occasionally, plerocerci were found in the musculature and in the gill arches (Fig. 7), although there was no systematic search of such sites for plerocerci. Merocerci of *Molicola horridus* occurred in the livers of a limited number of species of teleosts, but the intensity of infection was high and the infections were readily observable at autopsy (Fig. 6).

Species of larval trypanorhynch cestodes found in both teleost (as larvae) and elasmobranch (as adult) hosts at sites along the GBR and off NC are shown in Tables 1 and 2.

From the GBR, the specimens examined were obtained from the dissection of more than 9000 fish, although not all were specifically examined for trypanorhynch cestodes. Likewise, from NC, approximately 3800 fish were examined but the body cavity was not examined in every fish, as explained by Justine et al. [21–23]. Consequently, prevalence data were available for some species only and abundance data were not available; for most species only presence-absence data were available (with one exception from Lizard Island).

No trypanorhynch metacestodes were found in the families Blenniidae (*n* = 215), Chaetodontidae (*n* = 1638), Gobiidae (*n* = 183), Kyphosidae (*n* = 30) and Scaridae (*n* = 147) from the GBR. Likewise, no metacestodes were found in the families Atherinidae (*n* = 13), Apogonidae (*n* = 19), Echeneidae (*n* = 10) and Haemulidae (*n* = 10) in NC. In addition, although the families Serranidae, Lethrinidae and Lutjanidae were frequently infected with trypanorhynch metacestodes, this pattern was not uniform across all species within these families and in NC, no trypanorhynch metacestodes were found in *Epinephelus areolatus* (*n* = 12), *E. merra* (*n* = 18), *Lethrinus atkinsoni* (*n* = 12), *L. nebulosus* (*n* = 14), *Lutjanus fulviflamma* (*n* = 10) and *Lu. kasmira* (*n* = 14).

Members of the Tentacularioidea differ from other trypanorhynch metacestodes as they are present as plerocercoids (= post-larvae) rather than plerocerci [14] and may be found in intestinal contents as well as in the viscera. In New Caledonia, tentacularioids were frequently found in smaller schooling fishes, often being the only trypanorhynchs encountered in these fishes.

In total, 33 named species were found (Tables 1 and 3) as well as three species of tentaculariid cestodes to which no current name could be applied. Lacistorhynchoid and tentacularioid trypanorhynchs dominated the fauna in terms of numbers of species recovered (Table 3), with the otobothrioid and gymnorhynchoid trypanorhynchs being less numerous.

**Table 3.** Summary of the fully identified taxa of larval trypanorhynch cestodes found in teleost fishes from the Great Barrier Reef and from New Caledonia.

Order	Total number of species	Great Barrier Reef	New Caledonia	Number of shared species (%)
Gymnorhynchoidea	5	5	1	1 (20%)
Lacistorhynchoidea	14	10	12	8 (57%)
Otobothrioidea	6	3	5	2 (33%)
Tentacularioidea	9	6	7	5 (55%)
All orders	33	22	23	15 (45%)

Prevalence data were obtained from 182 fish from various families collected during a single collecting trip to Lizard Island. The prevalence of trypanorhynch larvae was: 4/6 (77%) in scombrids, 5/7 (71%) in lethrinids, 2/13 (15%) in lutjanids, 8/9 (89%) in serranids and 1/109 (0.9%) in apogonids. Other fish families were represented by smaller numbers and were excluded.

### Tentacularioid metacestodes of uncertain identity

Superfamily Tentacularioidea Poche, 1926
Family Tentaculariidae Poche, 1926

1. *Nybelinia* sp. A (Fig. 8)

**Figures 8–11.**
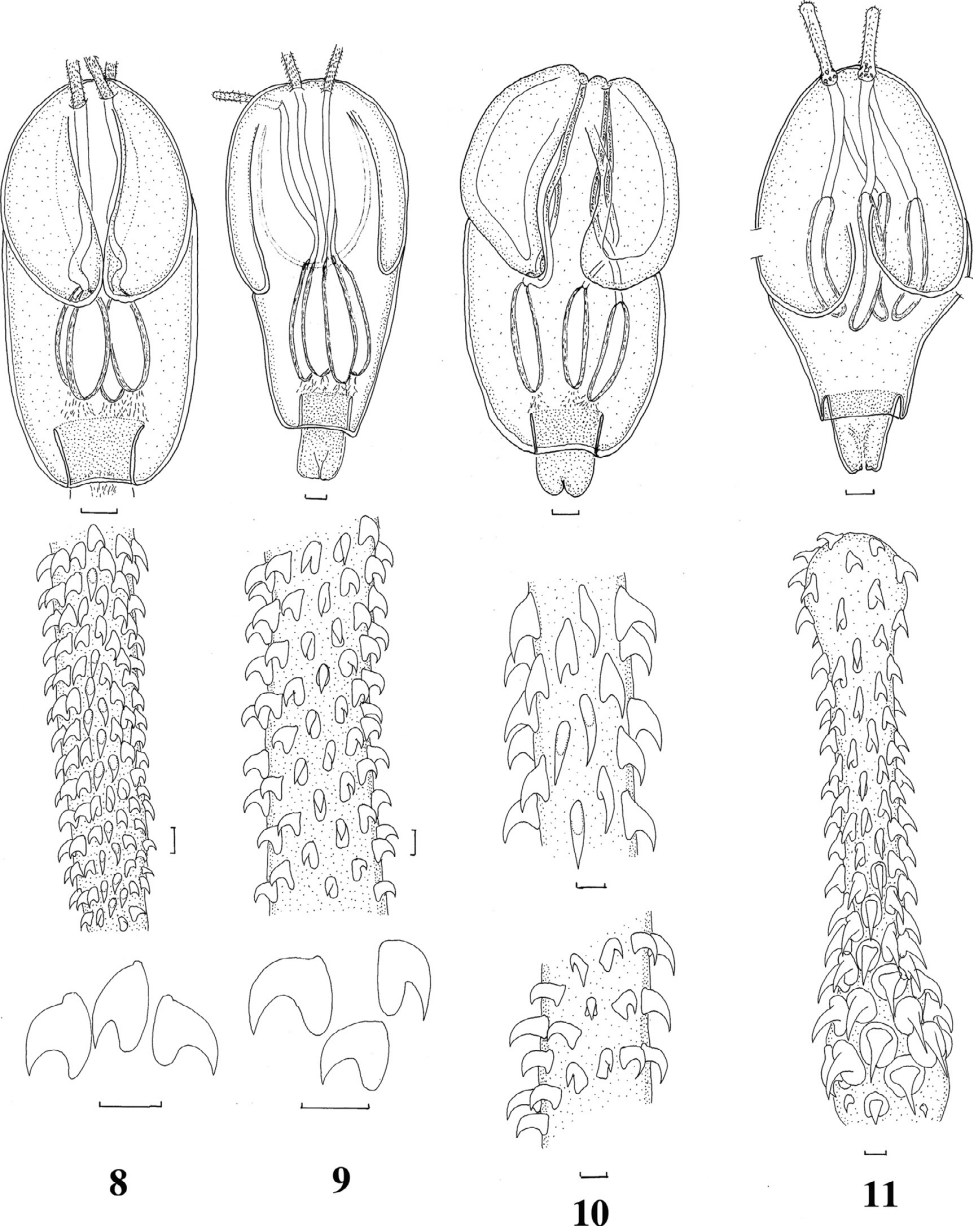
Tentacularioid metacestodes incompletely identified. **8.***Nybelinia* sp. A from *Herklotsichthys quadrimaculatus* (Rüppell, 1937). Scolex, basal and metabasal armature, hook profiles. Scale-bars: scolex and tentacle, 0.1 mm; hooks, 0.01 mm. **9**. *Nybelinia* sp. B from *Parupeneus multifasciatus* (Quoy & Gaimard, 1825). Scolex, basal and metabasal armature, hook profiles. Scale-bars: scolex and tentacle, 0.1 mm; hooks, 0.01 mm. **10**. *Heteronybelinia* sp. C from *Sufflamen fraenatus* (Latreille, 1804). Scolex, bothrial metabasal armature and antibothrial metabasal armature. Scale-bars: scolex 0.1 mm; hooks 0.01 mm. **11.***Nybelinia basimegacantha* Carvajal, Campbell & Cornford, 1976, specimen from *Neoniphon sammara* (Forsskål, 1775). Scolex, basal and metabasal armature. Scale-bars: scolex 0.1 mm; tentacle 0.01 mm.
  Material examined: plerocercoids from *Herklotsichthys quadrimaculatus* (Rüppell, 1937), New Caledonia, MNHN JNC2669C1, 2671A1.
  Scolex length 1200, pars bothrialis 580, pars vaginalis 520; bulbs ovoid, bulb length 250; velum 160; metabasal hooks: length 15, base 10.
  Remarks
  This species is similar to *N. queenslandensis*, but all measurements including those of the hooks are substantially smaller. In addition, the shape of the hooks differs (Fig. 8). The hook shape aligns the species with *N. lingualis* (Cuvier, 1817), *N. bisulcata* (Linton, 1889), *N. anthicosum* Heinz & Dailey, 1974 and *N. hemipristis* Palm & Beveridge, 2002, but *N. lingualis* and *N. bisulcata* differ in having much larger scoleces (2025–2700 and 2500, respectively) and bulbs (365–425 and 450–505, respectively) while the latter two species have much larger hooks (25–40). Consequently, these plerocercoids most closely resemble *N. lingualis* but cannot be assigned to this species with certainty.
2. *Nybelinia* sp. B (Fig. 9)
  Material examined: plerocercoid from *Parupeneus multifasciatus* (Quoy & Gaimard, 1825), New Caledonia, MNHN JNC2172 C4.
  Scolex length 1750, pars bothrialis 1100, pars vaginalis 1000, bulbs elongate, 560 long, velum 200, metabasal hooks: length 20, base 14.
  Remarks
  This specimen most closely resembles *N. strongyla* Dollfus, 1960 in scolex length, bulb length and hook size and shape, but differs in the length of the velum (690–830 in *N. strongyla* compared with 200 in the present material).
3. *Heteronybelinia* sp. C (Fig. 10)
  Material examined: plerocercoid from *Sufflamen fraenatus* (Latreille, 1804), New Caledonia, MNHN JNC3034.
  Scolex length 1440, pars bothrialis 770, pars vaginalis 680, bulbs elongate, bulb length 375, velum 125, metabasal hooks on antibothrial surface: length 17–19, base 8; on bothrial surface: length 25, base 18; basal armature heteromorphous.
  Remarks
  This specimen clearly belongs to *Heteronybelinia* as the hooks differ markedly in shape on the bothrial versus the antibothrial surfaces of the tentacle. Hook sizes are closest to *H. eureia* (Dollfus, 1960), but the specimen differs from this species in the number of hooks per half spiral and by the fact that in this specimen the bulbs are entirely posterior to the pars bothrialis while in *H. eureia*, they do not extend beyond the pars bothrialis. Therefore, this specimen cannot be accommodated within any known species of *Heteronybelinia*.
4. *Nybelinia basimegacantha* Carvajal, Campbell & Cornford, 1976 (Fig. 11)
  Material examined: plerocercoid from *Parupeneus multifasciatus* (Quoy & Gaimard, 1825), New Caledonia, MNHN JNC2111 C1; plerocercoid from *Neoniphon sammara* (Forsskål, 1775), New Caledonia, MNHN JNC2552.
  Specimen from *P. multifasciatus*: Scolex length 2600, pars bothrialis 1400, pars vaginalis 900, bulb length 1060, bulb width 130, velum 90.
  Specimen from *N. sammara*: Scolex length 1380, pars bothrialis 840, pars vaginalis 350; bulb length 450, bulb width 70, velum 70.
  Remarks
  Two specimens have been identified as belonging to this species with its characteristic armature. In spite of the fact that the armature of both specimens is identical, scolex measurements differed substantially and for this reason, the measurements of both specimens are presented. The specimen from *P. multifasciatus* although quite flattened, corresponds more closely with the original description of the species, also from *P. multifasciatus* from Hawaii [10]. In the specimen from *N. sammara*, all measurements are shorter but the tentacular armature is identical.

## Discussion

### General comments

Although the records of trypanorhynch infections listed here are based on the dissection of thousands of fish from both the GBR and NC, the data collected are based on opportunistic collecting and must be viewed in this light. Few prevalence or intensity data were collected and the data are based largely on the presence of trypanorhynch metacestodes. Fish examined that did not harbour metacestodes were not included in the data presented in the tables but representative examples have been indicated in the results.

In spite of these limitations, the large numbers of metacestodes collected from both regions provide a significant basis for comparing trypanorhynch metacestodes of teleosts inhabiting coral reefs.

Several features are evident from the data presented. In spite of potential differences in the fish faunas between the two regions examined and possible biases in sampling approaches, an extremely large number of fish specimens (thousands) was examined at each locality and even though the methods of examination varied to some degree, the study encompassed a wide range of fish families at both sites. Overall, 45% of the trypanorhynch species recorded here occurred in both regions. In addition, the trypanorhynch species most commonly encountered were similar in both locations. Records of adults from elasmobranchs from both of these regions provided additional information on potential life cycles and the collection included numerous new host and geographical records.

### Host specificity

Notwithstanding the opportunistic nature of the collecting, several aspects of host specificity are detectable within the data set and are worthy of discussion particularly since Palm & Caira, 2008 [30] have shown that specificity of the larval stages of trypanorhynchs is generally lower than that of the adults. First, it is evident that several fish taxa were rarely infected with trypanorhynchs. Thus, despite examination of substantial numbers of Blenniidae, Chaetodontidae, Gobiidae, Kyphosidae and Scaridae, no trypanorhynchs were found in these taxa. Other taxa strikingly underrepresented, though heavily sampled, were the Acanthuridae, Pomacentridae and Echeneidae. We do not suggest that these taxa have been exhaustively examined, but certainly they are depauperate relative to families such as the Balistidae, Lethrinidae, Scombridae and Serranidae.

Among the teleost fishes that were infected, there was evidence of both stenoxenicity (parasitism of closely related species) and euryxenicity (parasitism of distantly or ecologically related species). In the stenoxenous category, *Molicola horridus* was seen in two species of Diodontidae, *Pterobothrium australiense* has been seen only in labrids (one record), *Pseudogilquinia microbothria* was found only in lethrinids (both in NC and the GBR) and *Dasyrhynchus basipunctatus* occurred overwhelmingly in tetraodontiforms (five species) although also once in a fistulariid. The apparently restricted distributions of such species are doubtless subject to refinement with further collecting but it seems highly unlikely that they will prove to be euryxenous in the same way as are some other species.

We detected some evidence of the absence of trypanorhynch species in particular fish groups. The best evidence comes from the family Serranidae which is probably the most thoroughly characterised for its trypanorhynch fauna. The serranid fishes collected tend to be large and easily examined for trypanorhynchs with which they are often heavily infected. Our results incorporate reports from 25 serranid species and of the 181 host/parasite combinations detected, 55 were from serranids; the next highest number of combinations came from the Lethrinidae with 14. The extent to which the characterisation of this family is comprehensive is demonstrated by the fact that six of the ten trypanorhynch species recorded in this family have been reported from more than one serranid species; three species were found in ten or more serranid species although four species were found in only one. We infer that the true trypanorhynch richness is thus not likely to be very much greater than the 10 species reported so far in this region. Thus, we predict that species that have been reported relatively frequently in other fishes are genuinely absent, rather than have simply not yet been collected. Most striking in this respect are the species of the Tentacularioidea. Twelve species of this superfamily are reported here in 34 host/parasite combinations, but none in serranids. The apparent absence of a range of species from the Serranidae thus appears consistent with the high host specificity seen for the species described above.

Several species showed remarkably low specificity. Thus, *Callitetrarhynchus gracilis* was reported here from five fish orders and 18 families, *Floriceps minacanthus* from two orders and six families, *Pseudotobothrium dipsacum* from three orders and six families, *Pseudolacistorhynchus heroniensis* from two orders and four families and *Pseudolacistorhynchus shipleyi* from three orders and five families. The absence of any detectable specificity in these species leads to the prediction that further sampling will lead to even larger host ranges for these species.

*Callitetrarhynchus gracilis* exhibited the widest host range and has a cosmopolitan distribution [27] with carcharhinid sharks as its primary definitive hosts in the Australian region [1]. Currently recorded in the intermediate stage from approximately 130 species of teleosts [16, 27, 29], 23 new host records have been added in the present study.

*Floriceps minacanthus* appears to be limited to the Indo-Pacific region, and again, its known definitive hosts are carcharhinid sharks [26], with adults having been reported from four species of *Carcharhinus*. However, the present record in *Triaenodon obesus* is the first from a shark not belonging to this genus. Plerocerci have been reported from 13 species of teleosts [27, 29] from the Red Sea, Australia and off Indonesia and Hawaii while 14 new species of teleosts are reported here as hosts.

*Pseudotobothrium dipsacum* was also found in a wide variety of teleosts. It has previously been reported from numerous species of teleosts ranging from the west coast of Africa to Australia [4, 27]. Eight new hosts, all from New Caledonia, have been added in the present study. In spite of its wide host range and distribution, its definitive hosts remain unknown.

*Pseudolacistorhynchus heroniensis* is known only from the GBR and from NC but is found in a wide range of teleosts, with 12 new teleost hosts being added in the current study. The only record of the adult parasite is a single collection from *Stegostoma fasciatum* from New Caledonia [6]. The specimens collected were either immature or hyperapolytic such that some doubt exists as to whether this is the usual definitive host species.

*Pseudolacistorhynchus shipleyi* occurs widely in the Indo-West Pacific, with the adults being found in *Nebrius ferrugineus* off Sri Lanka [2]. In the current study, eleven new intermediate host records are reported.

The above five species occurred in a wide variety of teleost hosts with serranids (25 species), carangids (5), balistids (5), scombrids (5) and sphyraenids (5) being most frequently encountered. The same five species of trypanorhynch were the most commonly encountered species both on the Great Barrier Reef and off New Caledonia in spite of obvious differences in the species of fish infected at the two localities. There was no intentional bias in collecting activities, but it may have been that more of these larger fishes were collected than other smaller taxa.

Other species of trypanorhynch had a more restricted host distribution. Limited data on prevalence based on a single series of collections from Lizard Island suggested that trypanorhynch larvae were prevalent in larger fishes (serranids, sphyraenids, scombrids, lutjanids) but in small fish (a single family, Apogonidae) they occurred at a very low prevalence. However, these data were based on a very small sample of fish and need to be interpreted with caution.

Overall, the patterns of host specificity seen here, a mixture of stenoxenicity and euryxenicity, resemble that reported by Chambers et al., 2000 [11] for tetraphyllidean (*sensu lato*) metacestodes of GBR fishes. In that study, metacestode Type 4 was found in two orders and 12 families, whereas Types 9 and 10 were found only in labrids. However, in the study of tetraphyllidean metacestodes it is often not possible to be confident that a single morphotype represents only one species whereas the complex morphology of trypanorhynch scoleces makes identification to species quite reliable.

### Biogeography

Of the 33 trypanorhynch species reported here, 15 (45%) were found both in NC and on the GBR. Almost certainly this number underestimates the level of sharing between the two areas. Noticeably, the nine species reported in the largest number of host/parasite combinations were all found at both sites. Of the 21 species found in only one or two host/parasite combinations, only one (*Molicola horridus*) was found both in NC and on the GBR. It seems likely, or at least possible, that some species are restricted to one or other of the two sites but at present the evidence is generally marginal in this respect. The only robust parasitological study of which we are aware that has previously compared parasites of NC and the GBR is that of McNamara et al., 2012 [26] who analysed monorchiid trematodes of chaetodontids from NC and the GBR (as well as other sites in the Tropical Indo-West Pacific [TIWP]). Thirteen species of *Hurleytrematoides* Yamaguti, 1953 were found in total for the two sites of which just six were found at both sites for a similarity of 46%; four species were found only from the GBR and three only from NC. In every case, hosts suitable for the species not found in each area had been examined in numbers sufficient to suggest that they would have been found if present. The proportion of monorchiid species shared (46%) is thus remarkably similar to that for the trypanorhynchs. Given the much stricter specificity of monorchiids of chaetodontids (none known convincingly other than from chaetodontids) than of trypanorhynch metacestodes in general, we predict that further sampling for trypanorhynchs will see the levels of sharing increase.

Of the species found, eight (*C. gracilis*, *F. saccatus*, *Gr. exile*, *Hep. trichiuri*, *Het. estigmena*, *M. horridus*, *N. goreensis*, *O. penetrans*) have a cosmopolitan distribution, based on records in Palm, 2004 [27], while ten species are widely distributed in the Tropical Indo-West Pacific (TIWP) (*D. pacificus*, *F. minacanthus*, *N. basimegacantha*, *N. indica*, *Psgi. microbothria*, *Psgi. pillersi*, *D. basipunctatus*, *Psl. shipleyi*, *Psd. dipsacum*, *Pt. acanthotruncatum*). By contrast, seven species occur only in south-east Asia and Australasia (*N. queenslandensis*, *O. alexanderi*, *O. parvum*, *Psl. heroniensis*, *Psl. nanus*, *Pt. australiense*, *S. tigaminacanthus*). Several additional species (e.g. *Pt. lintoni*) with few, highly disjunct records are difficult to categorise. Nevertheless, with many of the trypanorhynch species encountered having extremely wide geographical distributions [31], it was not surprising that the species found on the GBR and from NC were broadly similar.

### Localisation in host

Apart from potential differences in the species of fish present at the two sites studied, or their abundance and hence ease of obtaining a particular species, other factors may be involved such as the location of trypanorhynch metacestodes in the body of the teleost. Most are found in the body cavity and are easily recognised. However, the metacestodes of *Gr. exile* occur only in the gill arches of *Sc. commerson* [35] and this site is not always examined for the presence of metacestodes. Similarly, the metacestodes of *Psg. microbothria* cluster around the oesophagus of *L. nebulosus* (unpublished) while those of *Pt. lintoni* are found in the musculature (unpublished). Failure to examine sites other than the body cavities may lead to differences in the species recovered.

### Life cycles

Combining the data obtained here with that available for adult trypanorhynchs in elasmobranchs in the same region has provided some insights into life cycles such as finding the adult of *Pt. acanthotruncatum* for the first time in *Pristis zijsron*. In addition, the definitive host range of *F. minacanthus* is expanded to include the shark *Triaenodon obesus*. Many life cycles remain to be identified, but broad scale collecting, such as that undertaken in this study, can be useful in identifying both potential intermediate and definitive hosts.

Species of *Diodon* warrant a particular mention as they are parasitised by several well-recognised trypanorhynch species including *Floriceps saccatus* and *Molicola horridus*. Infections with the latter species are particularly striking as much of the hepatic parenchyma may be replaced by metacestodes (Fig. 5). Species of *Diodon* are not only highly toxic [36], but can also inflate their bodies when threatened. As adults of these cestodes are found in large sharks such as *Prionace glauca* (Linnaeus, 1758) (see Dollfus, 1942) [14], it is tempting to assume that only large sharks are able to consume species of *Diodon*. Alternatively, it may be that the life cycles of these cestodes are completed using alternative intermediate hosts and their presence in species of *Diodon* indicates an occurrence in “dead-end” hosts. By comparison, in a study of the larval anisakid nematodes of teleosts off Lizard Island, Jabbar et al., 2012 [18] found no larval anisakids in their sample of tetraodontiform fishes, which would potentially be “dead-end” hosts for these nematodes.

## Conclusion

This is the first study to attempt to examine the trypanorhynch larval cestode fauna of coral reef teleosts in the west Pacific, examining reefs on the GBR and NC. The trypanorhynch fauna was dominated numerically by a small number of species at both sites with considerable similarity between the two localities examined. Although large numbers of teleosts were examined at both sites, it is most unlikely that the trypanorhynch fauna has been exhaustively surveyed and more detailed comparisons must await much more extensive sampling. Nevertheless, apart from characterising the general features of the fauna, this study has provided additional insights into host specificity and life cycles of these cestode parasites.

## Conflict of Interest

The Editor-in-Chief of Parasite is one of the authors of this manuscript. COPE (Committee on Publication Ethics, http://publicationethics.org/), to which Parasite adheres, advises special treatment in these cases. COPE wrote: “Editors should not be denied the ability to publish in their own journal, but they must not exploit their position. The journal must have a procedure for handling submissions from the editor or members of the editorial board that ensures that peer review is handled independently of the author/editor. This process should be detailed once the paper is published.” In this case the peer-review process was handled by Invited Editor Dominique Vuitton.
